# Editorial: Advancing cartilage regeneration and repair: biomaterials and biomechanical strategies

**DOI:** 10.3389/fbioe.2026.1947424

**Published:** 2026-09-08

**Authors:** Silvia Todros, Alice Berardo, Cristina Cavinato, Paolo Gargiulo

**Affiliations:** 1 Department of Industrial Engineering, University of Padova, Padova, Italy; 2 Laboratoire de Mécanique et Génie Civil (LMGC), University of Montpellier, Montpellier, France; 3 The Institute of Biomedical and Neural Engineering, School of Science and Engineering, Reykjavík University, Reykjavík, Iceland; 4 Department of Science, University Hospital Landspitali, Reykjavík, Iceland

**Keywords:** biomechanical properties, bioprinting, cartilage repair, extracellular matrix, hydrogel, polymeric biomaterial, tissue engineering

Cartilage replacement and regeneration remain major challenges in surgery and tissue engineering due to the limited capacity for self-repair of this avascular tissue with low cellular content. The major causes of articular cartilage degeneration include aging-related changes, chronic mechanical stress, joint instability, and inflammatory processes, which contribute to extracellular matrix degradation and the progression of osteoarthritis. To address these pathological conditions, recent research has focused on developing biomaterials that replicate the structural, biological, and mechanical properties of native cartilage. Among these, hydrogels and other natural or synthetic polymers have shown promising results due to their similarity to the extracellular matrix and their ability to support tissue regeneration.

Advancing effective repair strategies requires bridging the gap between understanding healthy and diseased cartilage and developing biomimetic materials capable of restoring tissue function. Successful cartilage regeneration depends on elucidating the structure–function relationships of native and pathological tissue and translating these insights into biomaterials and biomechanical strategies.

In this context, Santschi et al. demonstrated how detailed analysis of cartilage morphology and surface architecture, especially in the rarely studied acetabular labrum, can provide design criteria for next-generation biomimetic grafts. Using an animal model, the authors characterized surface microstructure, lubricin distribution, compressive stiffness and friction, and translated these findings into the design of biomimetic fibrous scaffolds. This work emphasizes that regenerative approaches should mimic the structural organization underlying tissue mechanical behavior.

Beyond structural organization, cartilage function critically depends on its tribological behavior. Consequently, methods capable of accurately characterizing friction on anatomically realistic joint surfaces are essential for understanding tissue physiology and degeneration. To address this need, de Roy et al. developed an innovative pin-on-surface tribometer equipped with a six-degree-of-freedom robotic arm. The experimental setup was first validated on synthetic polymers and then used to evaluate the frictional properties of tibial plateau cartilage in an animal model. A key strength of this platform is the ability to characterize curved anatomical surfaces and identify localized defects that are difficult to assess with conventional tribological systems.

While tribological analyses characterize cartilage surface function, successful evaluation of osteochondral repair requires quantitative assessment of the mechanical behavior of the entire grafted tissue. Complementing tribological characterization, Hernández Lamberty et al. introduced an advanced MRI-based methodology to quantify full-volume human patellar cartilage deformation before and after osteochondral allograft transplantation. Their results showed localized strain changes near the graft interface, suggesting that geometric mismatch alters the local mechanical environment. These findings demonstrate the value of functional mechanical metrics for evaluating cartilage repair beyond conventional morphological assessment.

Mechanical characterization alone, however, cannot fully explain cartilage degeneration. Understanding the molecular mechanisms linking mechanical loading to cartilage degeneration is equally important for guiding therapeutic innovation. Wang et al. showed that excessive mechanical loading activates a cellular pathway that promotes cartilage degeneration and identified a potential therapeutic target to prevent tissue damage. By elucidating mechanotransduction pathways involved in the osteoarthritis progression, these findings provide new insights into the relationship between mechanical loading and cartilage health, with potential implications for future osteoarthritis treatments.

Bridging experimental characterization and engineering design requires robust physical models. Using patient-specific anatomical reconstruction and multi-material three-dimensional printing, Coato et al. developed a synthetic tibial plateau with spatially graded mechanical properties and validated its mechanical response against native articular cartilage through indentation testing. This anatomically and mechanically realistic model provides a reproducible platform for the investigation of cartilage biomechanics and for the standardized assessment of implants and regenerative therapies, while also accounting for tissue heterogeneity.

Together, these studies naturally lead to the second major theme of the Research Topic: biomaterials for cartilage repair and regeneration. Insights gained from structural characterization, mechanical testing, tribological analysis, and mechanobiological investigations provide the basis for designing biomaterials capable of restoring tissue function.

Among several approaches, hydrogels have emerged as promising biomaterials for cartilage repair and regeneration. In this context, the review by Chen et al. highlights recent advances in injectable biomaterials for osteoarthritis treatment, focusing on their design, functional properties, and therapeutic applications. The authors discuss natural, synthetic, and composite hydrogels, together with advanced cross-linking strategies and stimuli-responsive systems that enable controlled drug delivery, reduce inflammation, and limit cartilage degeneration.

Beyond conventional hydrogels, smart engineered biomaterials offer the additional advantage of adapting their properties in response to external stimuli. Among these, electrosensitive hydrogels can reversibly modify their swelling, shape, and mechanical properties in response to electrical stimulation, offering new opportunities for adaptive and controlled therapeutic strategies. Farooqi et al. reviewed computational approaches for predicting the behavior of these materials under electric fields, comparing different modeling frameworks, and highlighting their role in optimizing hydrogel design and stimulation protocols for cartilage tissue engineering.

Injectable hydrogels used for viscosupplementation can also be evaluated *in vitro* to assess their mechanical and tribological performance by testing them between artificial cartilage surfaces. Following this approach, Confalonieri et al. developed polyvinyl alcohol hydrogels for the tribological evaluation of hyaluronic acid-based gels. By comparing hydrogels with different polymer concentrations, they identified an artificial cartilage formulation that most closely matched the compressive and frictional behavior of human articular cartilage, providing a reproducible and physiologically relevant platform for evaluating viscosupplementation treatments under controlled conditions while reducing the need for biological specimens.

The ability to develop scaffolds that reproduce cartilage architecture with increasing precision is demonstrated by Buttner et al. Their study highlights the potential of bioprinting technologies to fabricate engineered tissues that progressively acquire structural and mechanical properties resembling those of native cartilage, exemplifying the transition from biomaterial design toward functional tissue biofabrication.

Because cartilage regeneration often requires simultaneous restoration of the underlying subchondral bone, osteochondral repair remains a critical challenge. Schröter et al. addressed this Research Topic by developing multifunctional scaffold systems capable of supporting tissue integration across the osteochondral interface. This work highlights the increasing recognition that successful regeneration must address the joint as an integrated system rather than focusing solely on cartilage.

Taken together, the contributions collected in this Research Topic illustrate the increasingly interdisciplinary nature of cartilage research and outline a roadmap for future developments of cartilage tissue engineering. Advanced characterization techniques provide insights into the structural and mechanical properties of the healthy tissue; studies of degeneration elucidate the mechanisms driving disease; biomechanical models and platforms provide tools for tissue analysis and biomaterials optimization; and advanced biomaterials, bioprinted constructs, and osteochondral scaffolds translate these insights into regenerative solutions. The common message emerging from these contributions is that effective cartilage repair relies on a comprehensive understanding of cartilage structure, function, and mechanobiology, providing the foundation for a continuous translational pathway toward clinically translatable tissue regenerative therapies.

